# Best Harvest Moment for Colon Cancer Cells From 2D Cultures for 3D Tumoroid Bioprinting: An Experimental Observational Study

**DOI:** 10.7759/cureus.94367

**Published:** 2025-10-11

**Authors:** George Stancu

**Affiliations:** 1 Gastroenterology and Hepatology, Valahia Medical Center, Ploiești, ROU; 2 Internal Medicine, Fundeni Clinical Institute, Bucharest, ROU; 3 Faculty of Biology, University of Bucharest, Bucharest, ROU

**Keywords:** 3d-bioprinting, 3d-bioprinting tumors, cell and molecular biology, colon cancer, colon cancer treatment, gastroenterology, materials used in 3d bioprinting, oncology, personalised cancer treatment, printess bioprinter

## Abstract

Three-dimensional tumoroids promise to enhance personalized oncology by recapitulating many features of the tumor microenvironment. In this study, human colon cells from biopsy specimens were expanded in 2D cultures and subsequently analyzed for viability, morphology, and cell counts using phase-contrast microscopy combined with FIJI ImageJ processing. Notably, survival among patient-derived cultures was variable: Patients 1 and 2 cells did not survive past Day 1; Patient 3 cells survived for nine days; Patient 4 cells, for four days; Patient 5 cells survived for 28 days - with cells seeded to a second flask (designated 5S1) on Day 9 that survived for 20 days - and Patient 6 cells survived for 17 days. Our observations indicate that only cultures with extended survival (notably Patients 5 and 6) maintain the density and morphological uniformity required for optimal bioink formulation and subsequent bioprinting of tumoroids. According to the data obtained, the best day for harvesting cells for mixing with bioink for bioprinting is the ninth day.

## Introduction

Reliable in vitro models for colorectal cancer require not only robust cell proliferation but also sustained cell viability over time. Tumoroids, which mimic the three-dimensional architecture of tumors, depend on high-quality input cells [1]. In our workflow, cells isolated from colon biopsies are first expanded in 2D cultures and then incorporated into bioink for 3D bioprinting [2]. An important challenge is that patient-derived cells can vary significantly in their ability to survive under standard conditions. In our study of six patients, only a subset yielded long-term cultures: while Patients 1 and 2 cells degenerated by Day 1, cells from Patients 3 to 6 exhibited variable survival times - with Patient 5 demonstrating the longest survival. Importantly, on Day 9, Patient 5 cells were split into a second flask (5S1) and monitored separately, revealing intra‐patient variability in survival. Phase‐contrast microscopy images and subsequent FIJI processing provided both qualitative and quantitative data to determine the optimal parameters for 3D tumoroid bioprinting [3-7].

## Materials and methods

Ethics statement

All procedures involving human participants and their tissues were conducted in accordance with the ethical standards of the institutional and national research committee, and with the 1964 Helsinki Declaration and its later amendments. The study protocol was reviewed and approved by the Fundeni Clinical Institute Ethics Committee (approval no. 7445). Prior to colonoscopy, written informed consent was obtained from each participant for both the collection of biopsy specimens and the subsequent open-access publication of anonymized data. All data processing and handling activities complied with the European Union's General Data Protection Regulation (GDPR (EU 2016/679)).

Patient cohort and sample collection

Colonic mucosal biopsy specimens were obtained from six patients undergoing routine diagnostic colonoscopy at the Valahia Medical Center in Ploiești, Romania. No additional risks were posed to the patients, as samples were collected during a clinically indicated procedure. Each biopsy specimen measured less than 5 mm in size. Immediately after collection, samples were placed in a sterile container with Dulbecco's Phosphate-Buffered Saline (DPBS), supplemented with 100 U/mL penicillin and 100 µg/mL streptomycin (Antibiotic-Antimycotic; Thermo Fisher Scientific, Waltham, MA, USA), to prevent microbial contamination. Samples were transported in a portable incubator at 37°C to the laboratory for processing, with a maximum transport time of two hours [7,8].

Tissue processing and primary cell isolation

Upon arrival in the laboratory, biopsy specimens were processed immediately. Tissues were washed three times in sterile DPBS to remove residual blood and contaminants. Using sterile surgical blades, the tissues were meticulously minced into approximately 1 mm³ fragments to increase the surface area for enzymatic digestion. The fragments were then transferred to a digestion solution consisting of 0.25% trypsin-EDTA (Thermo Fisher Scientific), dissolved in DPBS. Enzymatic digestion was carried out in a shaking water bath at 37°C for 10 minutes to dissociate the tissue and liberate individual cells [9].

The resulting cell suspension was filtered through a sterile 100 µm nylon cell strainer (Falcon) into a 50 mL conical tube to remove undigested tissue debris and large aggregates. The filtered suspension was centrifuged at 300 × g for 10 minutes at room temperature. The supernatant was carefully aspirated, and the cell pellet was resuspended in a complete growth medium for subsequent counting and seeding.

Two-dimensional cell culture and maintenance

The isolated primary cells were seeded at an initial density of approximately 50,000 cells/cm^2^ into T-25 culture flasks (Corning Incorporated, Corning, NY, USA). The growth medium consisted of Dulbecco’s Modified Eagle Medium/Nutrient Mixture F-12 (DMEM/F12; Thermo Fisher Scientific), supplemented with 10% fetal bovine serum (FBS; Thermo Fisher Scientific), 2 mM L-glutamine, and 1% penicillin-streptomycin. Cultures were maintained in a humidified incubator at 37°C with a 5% CO_2_ atmosphere [10].

The culture medium was replaced with fresh, pre-warmed complete medium every 48 hours to ensure nutrient replenishment and waste removal. Cell morphology, attachment, proliferation, and any signs of senescence (e.g., vacuolization and enlargement), or detachment were monitored daily using an inverted phase-contrast microscope (OpenFlexure Microscope v7).

Subculture and propagation

To assess the impact of serial passaging on cell viability and proliferative capacity, cells from Patient 5 were subcultured upon reaching approximately 80% confluence (on Day 9). Briefly, the spent medium was aspirated, and the cell monolayer was gently rinsed with phosphate-buffered saline (PBS, pH 7.4) to remove residual serum, which can inhibit trypsin activity. Cells were detached using 2 mL of 0.05% trypsin-EDTA solution for five minutes at 37 °C. Enzymatic activity was neutralized by adding an equal volume of complete growth medium, containing FBS [11].

The cell suspension was collected, centrifuged at 300 × g for five minutes, and resuspended in fresh medium. Cells were reseeded at a 1:2 split ratio into a new T-25 flask, designated “Patient 5S1” (first subculture). Both the original (P5) and subcultured (P5S1) flasks were maintained under identical culture conditions, with the same feeding schedule.

Phase-contrast microscopy and time-course imaging

Longitudinal monitoring of cell growth was performed via phase-contrast microscopy at predefined time points (Days 1, 2, 4, 7, 9, 14, 17, 20, 22, 23, 26, and 28). For each flask and time point, imaging was conducted using an Openflexure microscope v7 inverted microscope, equipped with a 10× objective lens and a Raspberry Pi digital camera. To ensure consistency and minimize observational bias, three non-overlapping, representative fields of view were captured per flask. Microscope settings, including lamp intensity and exposure time, were standardized and kept constant throughout the entire study to guarantee comparable image quality for quantitative analysis [6,12].

Image processing and quantitative analysis

All quantitative image analysis was performed using FIJI (distribution of ImageJ, version 1.53c) [3,13]. A standardized, automated processing workflow was applied to all images to ensure objectivity and reproducibility: (i) images were converted to 8-bit grayscale; (ii) a rolling-ball background subtraction algorithm (radius = 50 pixels) was applied to correct for any uneven illumination; (iii) images were converted to binary masks using an automatic Otsu thresholding method; (iv) the "Watershed" segmentation algorithm was applied to separate adjacent or overlapping cells; and (v) the "Analyze Particles" function was used to quantify objects within a defined size range (50-2,000 µm²), to exclude debris and artifacts. (vi) The mean cell count from the three fields per flask was calculated for each time point, and results are presented as mean ± standard deviation (SD) [6].

Bioink formulation and 3D bioprinting

For 3D culture, cells harvested at the optimal time point were used. The bioink was formulated as a hydrogel blend of 2% (w/v) sodium alginate (Sigma-Aldrich; Merck KGaA, Darmstadt, Germany) and 1% (w/v) gelatin from porcine skin (Sigma-Aldrich), prepared in sterile DPBS. The cell pellet was resuspended in the hydrogel precursor solution to achieve a final cell density of 1 × 10^6^ cells/mL.

Bioprinting was performed using a Printess Bioprinter without a temperature-controlled printhead and using a 25 G needle nozzle (inner diameter: 160 µm) [14]. The printing parameters were set as follows: extrusion pressure, 15 kPa; print speed, 10 mm/s; and layer height, 0.5 mm. The constructs were printed in a 5 × 5 mm grid pattern. Immediately after printing, the structures were crosslinked by immersion in a 100 mM calcium chloride (CaCl_2_) solution for five minutes to ionically crosslink the alginate component. The crosslinked constructs were then rinsed in DPBS and transferred to complete growth medium for culture in a 37°C, 5% CO_2_ incubator [4,15].

Statistical analysis

Quantitative data from image analysis are reported as mean ± SD. Given the exploratory nature of this study and the limited sample size (n = 6 primary cultures), formal inferential statistical testing between patients was not conducted. Instead, culture longevity and survival were visualized and compared using Kaplan-Meier survival curves, where an event was defined as the time point at which a culture exhibited no viable cells or conclusive senescence. Data analysis was performed with JASP software (version 0.16.3.0; JASP Team, University of Amsterdam, Amsterdam, the Netherlands).

## Results

Representative microscopy and FIJI-processed images

Table 1 shows examples from selected cultures. In each entry, the first image is the original color phase-contrast image, and the second image is the corresponding FIJI-processed (black and white) image with segmentation overlays. Note that, due to the limited survival times, representative images for Patients 1-4 were obtained at their final viable time points.

**Table 1:** Representative Microscopy Images Evolution

Patient	Culture Day	Flask Capture	Image Description
1	1	Single	Culture failed to establish; cells did not survive beyond Day 1.
2	1	Single	No cell adhesion observed; culture did not survive beyond Day 1.
3	9	Single	Limited survival; cells visible up to Day 9 with modest clustering.
4	4	Single	Culture survived only until Day 4; only sparse clusters were evident.
5	9	Patient 5	Pre-split culture; moderate clustering evident.
5	9	Patient 5S1	Post-split culture; denser clusters observed after seeding into the second flask.
6	17	Single	Cells maintained up to Day 17 with high confluence and uniform morphology, ideal for bioprinting.

FIJI cell count data adjusted for viability

In Table 2, we summarize the FIJI-derived cell count data for the patients with surviving cultures. Since Patients 1 and 2 did not survive, their data are not included in the further analysis. Note that each entry reflects the final count obtained before culture termination.

**Table 2:** FIJI Cell Count Summary for Surviving Cultures Note: Due to differences in survival times, cell counts are reported at the most representative viable time point for each patient culture.

Patient ID	Final Viable Culture Day	Flask Capture	Cell Count (FIJI)	Comments
3	9	Single	415	Modest proliferation; culture terminated at Day 9.
4	4	Single	509	Limited survival; reached confluence quickly but unstable.
5	28	Patient 5	1.185 (Day 5 value reported earlier)	Long-term culture; robust viability up to 28 days.
5S1	20	Patient 5S1	3.507	Post-split culture; maintained for 20 days with higher yield.
6	17	Single	17.691	Optimal confluence and morphological stability at termination.

Optimal harvest time and implications for bioprinting

**Figure 1:**
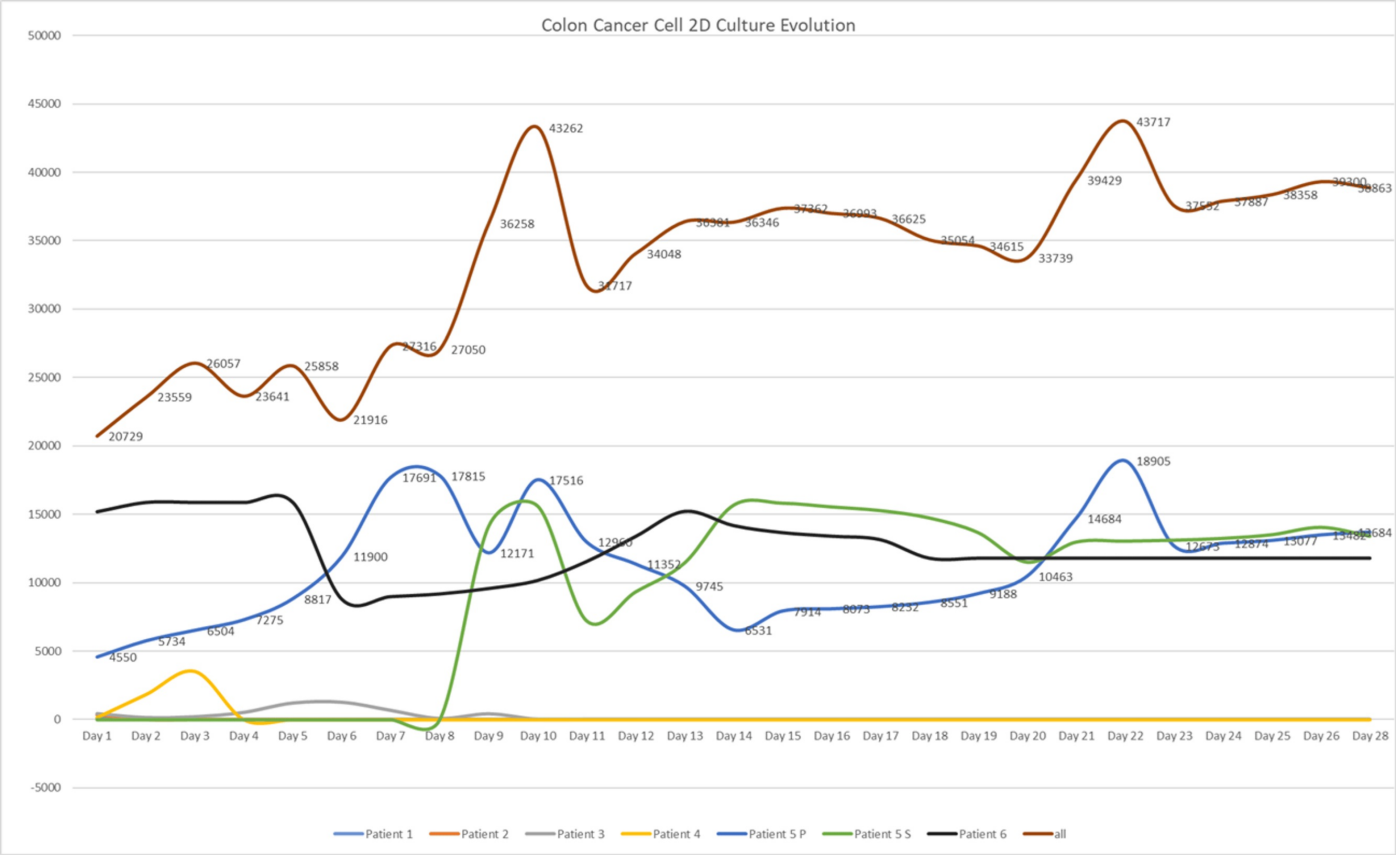
Colon Cancer Cell 2D Culture Evolution The cell count is presented from Day 1 to Day 28.

The combined imaging (both original and FIJI-processed) and cell count data demonstrate that only cultures with extended survival - specifically Patient 5 (and its split flask, 5S1) and Patient 6 - achieved the cell density and uniform morphology required for effective bioink formulation. In contrast, the short survival times of Patients 1-4 limit their use in downstream bioprinting applications (Tables 3-7). For Patient 5, although cells were monitored for 28 days in one flask, the split culture 5S1 (surviving 20 days) shows that intra-patient handling significantly affects cell yield and viability. Analyzing the data from all patients, the interval for harvesting cells for bioprinting tumoroids is between the 7th and 12th days of culture (Figure 1). The recommended day for harvesting is the ninth day after culturing. This timeframe generates the highest number of cells that can be transferred to 3D tumoroids.

**Table 3:** Analysis of the Cell Number for All Patients Daily Counted With Fiji Software

No.	Patient Label	Day 1	Day 2	Day 3	Day 4	Day 5	Day 6	Day 7	Day 8	Day 9	Day 10	Day 11	Day 12	Day 13	Day 14	Day 15	Day 16	Day 17	Day 18	Day 19	Day 20	Day 21	Day 22	Day 23	Day 24	Day 25	Day 26	Day 28
1	Patient 1	90	0	0	0	0	0	0	0	0	0	0	0	0	0	0	0	0	0	0	0	0	0	0	0	0	0	0
2	Patient 2	331	0	0	0	0	0	0	0	0	0	0	0	0	0	0	0	0	0	0	0	0	0	0	0	0	0	0
3	Patient 3	415	131	188	509	1185	1240	652	64	402	0	0	0	0	0	0	0	0	0	0	0	0	0	0	0	0	0	0
4	Patient 4	163	1835	3507	0	0	0	0	0	0	0	0	0	0	0	0	0	0	0	0	0	0	0	0	0	0	0	0
5	Patient 5	4550	5734	6504	7275	8817	11900	17691	17815	12171	17516	12960	11352	9745	6531	7914	8073	8232	8551	9188	10463	14684	18905	12673	12874	13077	13482	13684
6	Patient 5S	0	0	0	0	0	0	0	0	14118	15588	7216	9323	11430	15645	15796	15527	15258	14720	13644	11493	12962	13029	13096	13230	13498	14035	13396
7	Patient 6	15180	15859	15858	15857	15856	8776	8973	9171	9567	10158	11541	13373	15206	14170	13652	13393	13135	11783	11783	11783	11783	11783	11783	11783	11783	11783	11783
	All	20729	23559	26057	23641	25858	21916	27316	27050	36258	43262	31717	34048	36381	36346	37362	36993	36625	35054	34615	33739	39429	43717	37552	37887	38358	39300	38863

**Table 4:** FIJI Analysis for Patients 1-3

Patient	Day (Line P)	Cell Count (Line P)	Medium Size (µm)
1	1	90	52.11
2	1	331	49.625
3	1	415	51.72
3	2	131	51.466
3	3	188	45.46
3	4	509	40.34
3	5	1185	49.268
3	6	1240	49.6
3	8	64	30
3	9	402	35.55

**Table 5:** FIJI Analysis for Patient 4

Patient	Day (Line P)	Cell Count (Line P)	Medium Size (µm)
4	1	163	66.129
4	3	3507	38.96

**Table 6:** FIJI Analysis for Patient 5

Patient	Day (Line P)	Cell Count (Line P)	Medium Size (Line P)	Day (Line S1)	Cell Count (Line S1)	Medium Size (Line S1)
5	1	4550	43.225	-	-	-
5	2	5734	205.903	-	-	-
5	9	12171	185.261	0	14118	218.889
5	10	17516	127.612	1	15588	121.582
5	11	12960	141.899	2	7216	95.525
5	14	6531	49.225	5	15645	157.73
5	15	7914	219.995	6	15796	166.674
5	20	10463	81.543	11	11493	79.184
5	22	18905	99.63	13	14066	162.797
5	23	12673	178.621	14	12962	204.979
5	26	13482	151.466	17	14035	165.289
5	28	13684	167.369	19	13396	148.345

**Table 7:** FIJI Analysis for Patient 6

Patient	Day (Line P)	Cell Count (Line P)	Medium Size (µm)
6	1	15180	95.182
6	2	15859	56.607
6	5	15856	140.026
6	6	8776	242
6	11	11541	96.37
6	13	15206	157.221
6	14	14026	166.221
6	17	13135	180.202
6	19	11783	253.012

Summary and implications for bioprinting

**Figure 2:**
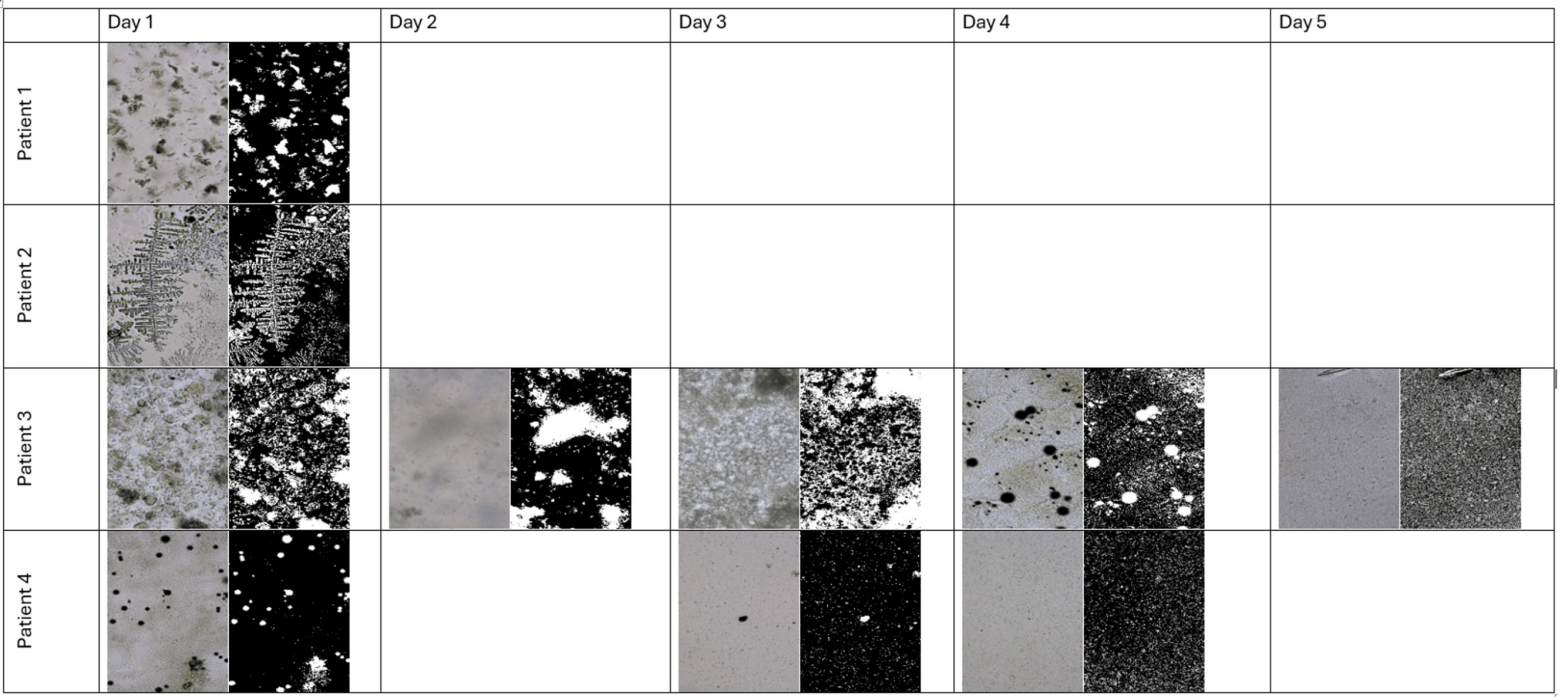
Microscopy and FIJI Images of the Flasks Containing Colon Cancer Cells of the Patients 1-4 From Days 1 to 5

**Figure 3:**
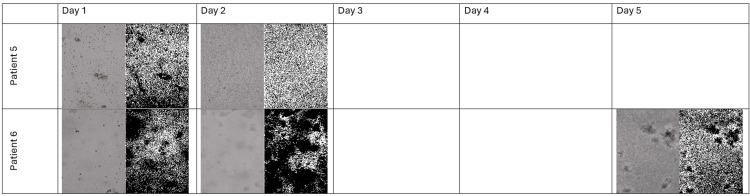
Microscopy and FIJI Images of the Flasks With Colon Cancer Cells of the Patients 5-6 From Days 1 to 5

**Figure 4:**
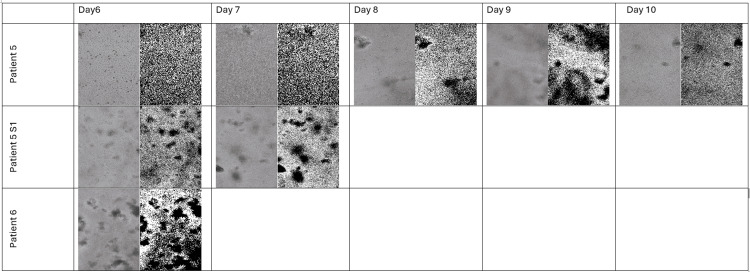
Microscopy and FIJI Images of the Flasks With Colon Cancer Cells of the Patients 5-6 From Days 6 to 10

**Figure 5:**
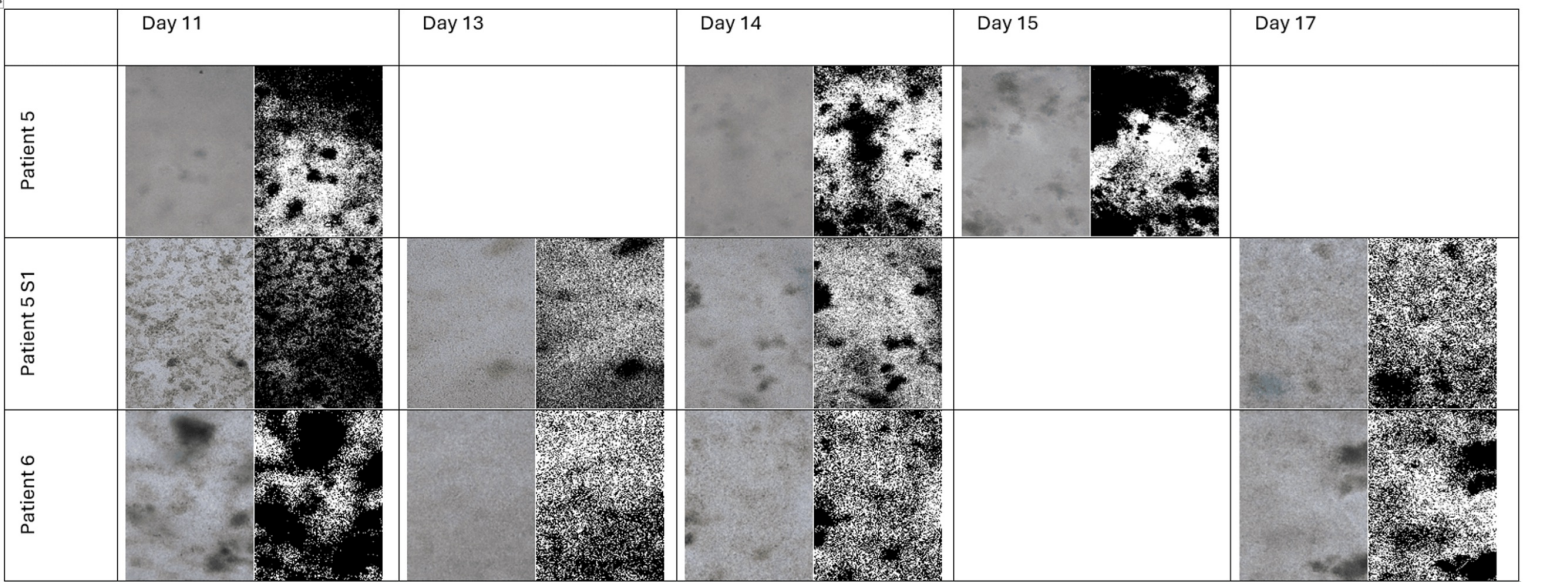
Microscopy and FIJI Images of the Flasks With Colon Cancer Cells of the Patients 5-6 From Days 11 to 17

**Figure 6:**
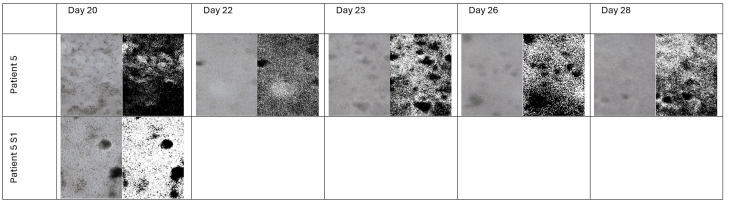
Microscopy and FIJI Images of the Flasks With Colon Cancer Cells of the Patients 5 and 5S1 From Days 20 to 28

The data below demonstrate that only Patients 3-6 produced cultures that survived beyond Day 1 (Figures 2-6).

In particular, Patient 5 (especially when considering both the primary and split cultures) and Patient 6 achieved extended survival and reached high cell counts with stable morphological profiles. These characteristics are crucial for generating reproducible bioinks and ensuring high print fidelity on the Printess Bioprinter for 3D tumoroid production. The detailed FIJI analysis - combining both quantitative cell counts and the corresponding mean size metrics - provides a solid foundation for selecting the optimal culture for downstream bioprinting applications.

Additional considerations

Future investigations could complement this FIJI-based analysis with additional markers (e.g., Ki-67 for proliferation or apoptosis markers) to further refine the criteria for long-term cell viability and optimal bioprinting outcomes. Moreover, standardizing culture handling protocols - to minimize intra-patient variability, as evidenced by the differences between Patients 5 and 5S1 - will be crucial for the scalability of this approach.

## Discussion

The successful biofabrication of patient-specific tumoroids is a promising avenue for personalized oncology, yet it remains technically challenging. A primary bottleneck is the generation of a sufficient number of viable, homogeneous cells from primary tumor isolates to serve as a bioink feedstock. Our study systematically evaluated the 2D expansion phase of primary human colon cancer cells to identify a critical harvest window, finding that only a subset of patient-derived cultures (Patients 5 and 6) provided the necessary combination of longevity and uniform morphology for subsequent bioprinting. This starkly contrasts with the rapid senescence observed in Patients 1-4, underscoring the significant inter-patient heterogeneity inherent in primary cancer cell culture [16]. Such variability is not unique to colorectal cancer; similar challenges in establishing stable, proliferative cultures from a limited number of patient samples have been documented in other malignancies, including glioma and pancreatic ductal adenocarcinoma, where genetic and epigenetic diversity underlie differential ex vivo behavior [17,18].

The inherent limitations of 2D culture for capturing tumor biology are well established. Comparative analyses have consistently demonstrated that 3D organoid or spheroid systems better preserve key aspects of the original tumor, including stromal interactions, phenotypic heterogeneity, and drug response profiles, by more accurately mimicking the in vivo microenvironment [19,20]. Seminal work by the Clevers group established that colorectal cancer organoids can retain stem cell properties and recapitulate the parental tumor’s genetic landscape over long-term culture - features often lost in traditional 2D monolayers [21]. Our finding that Day 9 post-isolation represents an optimal harvest window for bioprinting aligns with the fundamental principles of these organoid systems. This time point appears to represent a convergent period where cell density and viability are maximized, but prior to the onset of replicative senescence or excessive contact inhibition, which can compromise cellular fitness for 3D assembly [22].

Beyond mere viability, the physical properties of the cell suspension are paramount for extrusion-based bioprinting. The rheological properties of a bioink, particularly its viscosity and shear-thinning behavior, must fall within a narrow range to ensure reliable extrusion, shape fidelity, and high cell survival [23,24]. These properties are directly influenced by cell concentration, size, and the presence of aggregates. The superior performance of cells from Patients 5 and 6 at Day 9 likely reflects an ideal balance: a high cell density that contributes to bioink structural integrity, without the formation of large, irregular clusters that can cause nozzle clogging, inconsistent extrusion, and compromised print resolution [25].

Our data also highlight that intra-patient technical variability is a pivotal factor often overlooked in protocol optimization. The divergent outcomes between the primary Patient 5 culture (P5) and its first subculture (5S1) underscore how subtle manipulations - such as variations in trypsinization time, mechanical stress during detachment, or reseeding density - can dramatically alter subsequent cell yield and viability. This sensitivity necessitates rigorously standardized protocols. As emphasized in recent biofabrication guidelines, the adoption of automated cell counters for precise quantification and real-time viability assays (e.g., based on membrane integrity or metabolic activity) is crucial for reducing operator-dependent variability and improving inter-laboratory reproducibility [26,27].

Despite these insights, our study has several limitations. The small patient cohort (n = 6) and exploratory nature constrain the generalizability of the Day 9 recommendation. Future investigations must incorporate larger, multi-center patient cohorts to validate this harvest window across a broader genetic and clinical spectrum of colorectal cancer. Furthermore, a direct side-by-side comparison with established Matrigel-based colon organoid culture protocols would help delineate the relative advantages of each approach for specific downstream applications [28]. Most critically, confirming that the bioprinted tumoroids maintain the molecular and functional characteristics of the original tumor is essential. Future work must integrate functional assays, such as chemosensitivity profiling and RNA sequencing, to validate that the Day 9 harvest window preserves not just cellular viability, but also the transcriptional and phenotypic fidelity of the source malignancy [29].

Looking forward, several strategies could further enhance the fidelity and utility of bioprinted tumoroids. Incorporating molecular viability markers (e.g., Ki-67 for proliferation and cleaved caspase-3 for apoptosis) into the assessment pipeline would provide a more nuanced readout of cellular health beyond simple morphology and adherence [30]. Furthermore, advanced bioink formulations that incorporate tissue-specific extracellular matrix components (e.g., collagen I and laminin) can provide crucial biochemical and mechanical cues to support long-term 3D culture and function [28]. Finally, moving towards more complex multi-cellular models by co-culturing cancer cells with patient-derived cancer-associated fibroblasts (CAFs) or immune cells will be essential to recapitulate the tumor microenvironment and its role in therapy resistance [29,30]. By defining an optimal harvest window and situating it within this broader methodological landscape, our study provides a foundational roadmap for optimizing the critical 2D-to-3D transition in the development of robust, patient-derived cancer models for personalized drug screening and biological discovery.

Future considerations

Future work could correlate these survival and quantitative imaging data with molecular markers of viability and proliferation (such as Ki-67) to further refine the criteria for selecting optimal cultures for bioprinting. Moreover, exploring the influence of extracellular matrix components within the bioink may enhance the functionality and longevity of the resulting tumoroids.

## Conclusions

This study confirms that, among the examined patient-derived colon cell cultures, only those with extended viability - specifically Patient 5 (and its derivative 5S1) and Patient 6 - achieve the cell density and uniformity required for 3D tumoroid bioprinting. The detailed analysis, incorporating both qualitative microscopy and quantitative FIJI cell counts, highlights that survival time is a critical parameter. Cells from Patients 1 and 2 failed to survive even one day, while Patients 3 and 4 exhibited too-short a lifespan (nine and four days, respectively) to be useful. Thus, for applications with the Printess Bioprinter, obtaining robust 2D cultures (as seen with Patients 5 and 6) is essential for generating reproducible bioinks and high-fidelity tumoroid constructs.
